# Siglec-15 as a New Perspective Therapy Target in Human Giant Cell Tumor of Bone

**DOI:** 10.3390/curroncol29100605

**Published:** 2022-10-13

**Authors:** Mengke Fan, Guochuan Zhang, Mingfang Xie, Xinbo Liu, Qi Zhang, Ling Wang

**Affiliations:** 1 Department of Orthopedic Research Center, Third Hospital of Hebei Medical University, Shijiazhuang 050051, China; 2 Department of Orthopedic Oncology, Third Hospital of Hebei Medical University, Shijiazhuang 050051, China; 3 Department of Thoracic Surgery, Fourth Hospital of Hebei Medical University, Shijiazhuang 050010, China

**Keywords:** giant cell tumor of bone, Siglec-15, CXCL8, functional assays, KEGG analysis, GO analysis

## Abstract

The main features of a giant cell tumor of bone (GCTB) are frequent recurrence and aggressive osteolysis, which leads to a poor prognosis in patients. Although the treatment methods for a GCTB, such as scraping and resection, effectively inhibit the disease, the tendency toward malignant transformation remains. Therefore, it is important to identify new treatment methods for a GCTB. In this study, we first found high Siglec-15 expression in GCTB tissues, which was significantly associated with Campanacci staging and tumor recurrence. In Spearman’s analysis, Siglec-15 expression was significantly correlated with Ki-67 levels in tumor tissues. In vitro, the mRNA and protein levels of Siglec-15 were high in GCTB stromal cells (Hs737. T), and Siglec-15 knockdown inhibited the biological characteristics of GCTB stromal cells. The RNA sequencing results enabled a prediction of the downstream genes by using the Kyoto Encyclopedia of Genes and Genomes (KEGG), Gene Ontology (GO), and MCODE analyses, and the findings showed that CXCL8 was significantly regulated by Siglec-15 and might be a promising downstream target gene of Siglec-15. Therefore, Siglec-15 may be a potential immunotherapy target for a GCTB.

## 1. Introduction

A giant cell tumor of bone (GCTB) is a transitional tumor that easily transforms into a malignant tumor; this tumor often occurs near the knee joint and shows obvious features of recurrence and osteolysis [1,2,3,4,5]. In Asia, the incidence of a GCTB accounts for approximately 5% of primary bone tumors and approximately 20% of benign tumors [6,7]. A GCTB is mainly detected in young adults between 20 and 40 years old [8]. Adjuvants, including phenol and methyl methacrylate, have been applied in GCTB surgical treatments, but the recurrence rate is still high (25–50%) [9,10,11,12,13]. Presently, though Denosumab has dramatically changed the treatment paradigm for a GCTB, which will be highly efficacious and well tolerated, there is still a chance that osteonecrosis of the jaw and rebound hypercalcemia occurred in GCTB patients [14]. Thus, it is of a great significance to further develop efficient and safe therapeutic drugs for a GCTB.

In the GCTB microenvironment, mononuclear stromal cells are tumor cells that mainly participate in the GCTB malignant process [15,16]. For example, the activity of GCTB stromal cells is enhanced with p62 overexpression, whereas the stromal cells were inhibited with the knockdown of the p62 gene [17]. Leukocyte activating adhesion factor (ALCAM) ALCAM+ stromal cells have clear stem cell characteristics, which promote the proliferation, migration, and invasion of GCTB stromal cells [18]. The osteoclast-like multinucleated giant cells are main cells that mediate osteolytic effects in a GCTB. It was reported that the RANKL-RANK-OPG pathway is closely associated with the formation, differentiation and function of osteoclast-like multinucleated giant cells. The denosumab, a human monoclonal antibody against RANKL, was developed according to the main effect of the RANK/RANKL mechanism in the pathogenesis of a GCTB [14]. Many studies showed that RANKL overexpression by GCTB stromal cells can stimulate the monocytes with RANK to merge into osteoclast-like multinucleated giant cells, leading to the over-resorption of the bone in a pathological area [19]. This further elucidates the key role of stromal cells and the effectiveness of the targeted suppression of stromal cells in the GCTB.

Siglec-15, also known as CD33L3, is a special family member of Siglecs that regulates nonadaptive and adaptive immune responses [20,21,22]. Previous studies have shown that Siglec-15 plays important roles in osteolysis and remodeling, microbial infection, and tumor processes [23]. In the research of osteoporosis, scholars have found that the adapter proteins DAP12 on an osteoclast is an important auxiliary signaling for osteoclast maturation and one of the DAP12 ligands is Siglec-15 [24]. Ishida-Kitagawa et al. found that Siglec-15 mRNA transcription is upregulated in osteoclasts by NFAT2 (a transcription factor, which is activated by RANK) [25], suggesting that RANKL and Siglec-15 jointly promote osteoclast differentiation and osteolytic function through a mutual regulation and synergy, which may be the molecular basis of a combined targeted treatment for RANKL and Siglec-15. Hiruma and colleagues first identified SIGLEC15 as a gene highly expressed on GCTB in 2011 [26]. In recent studies by Wang et al., Siglec-15 mRNA was highly expressed in cancer tissues such as bladder cancer, kidney cancer, and lung cancer [27]. In vivo, high Siglec-15 expression promoted an obviously decreased mouse survival in a melanoma mouse model [27]. In addition, we previously found that high Siglec-15 levels in osteosarcoma were associated with lung metastasis of osteosarcoma and promoted the viciousness of osteosarcoma cells and enhanced the EMT and MMP-9 levels in vitro and in vivo [28]. Thus, whether Siglec-15 influences GCTB progression needs to be further investigated.

**Figure 1:**
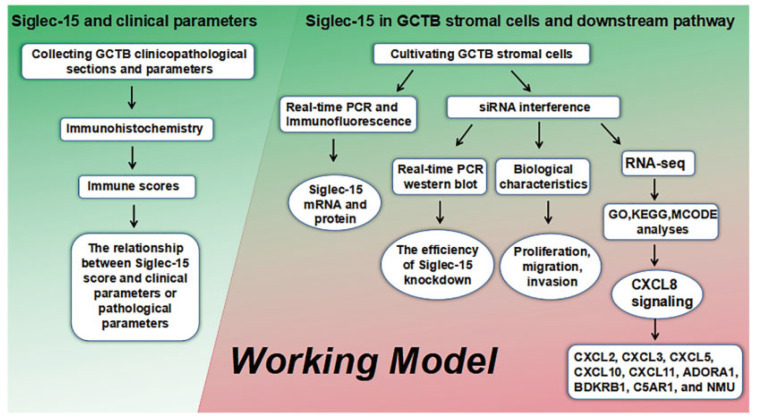
Working model illustrates the association between Siglec-15 and clinical parameters, the influence of high Siglec-15 for GCTB stromal cells, as well as the prediction of downstream pathways of Siglec-15 in GCTB stromal cells.

In this study, we first detected the levels of Siglec-15 in GCTB tissues and found that higher Siglec-15 levels were associated with Campanacci staging, tumor recurrence, and Ki-67 levels. In vitro, the knockdown of the Siglec-15 gene suppressed the growth, migration, and invasion of GCTB stromal cells (Hs737. T). We further predicted the downstream genes of Siglec-15 via RNA sequencing analysis. The Kyoto Encyclopedia of Genes and Genomes (KEGG), Gene Ontology (GO), and MCODE analyses showed that CXCL8 was a significant gene regulated by Siglec-15. In addition, the related genes of CXCL8 were predicted by a STRING analysis. The schematic diagram of work has been presented below (Figure 1).

## 2. Materials and Methods

### 2.1. Patient and Samples

Fifty-six section specimens of GCTB were collected from the Department of Pathology, the Third Hospital of Hebei Medical University, from 1 January 2012 to 31 December 2017. All sections were confirmed as GCTB tissues through clinical, imaging, and pathological diagnoses. None of the patients had undergone any radiotherapy, chemotherapy, denosumab treatment, or traditional Chinese medicine treatment. The detailed clinical parameters, including age, sex, tumor location, tumor size, Campanacci staging, and tumor recurrence, were collected. In addition, the pathological parameters, including CD163, CD34, CD68, and Ki-67, were collected from the pathology reports of GCTB patients (see Table 1 and Table 2). All of the experiments in this study were in accordance with the approved guidelines and regulations, and the experimental protocols were approved by the ethics committee of the Third Hospital of Hebei Medical University.

### 2.2. Cell Culture

The human GCTB stromal cells (Hs737. T) were purchased from the American Type Culture Collection (ATCC) and were then cultured in a high-glucose DMEM, containing 15% FBS and 1% penicillin/streptomycin, at 37 °C in a humidified 5% CO_2_ atmosphere [29]. When the GCTB stromal cell confluence reached 80–90%, the cells were digested by 0.25% trypsin-EDTA. The digestion ended with a complete medium addition when the cells became spherical under a microscope (at approximately 5 min). The cells were centrifuged at 1000 rpm for approximately 7 min and then divided into 2–3 flasks for subculture.

### 2.3. Antibodies and Major Reagents

The anti-Siglec-15 antibody (ab198684, rabbit, 1/100) was purchased from Abcam (Cambridge, UK) and the goat anti-rabbit IgG HRP was purchased from Zhongshan Golden Bridge Biotechnology (Beijing, China). The secondary antibodies for Western blotting or the immunofluorescence assays were purchased from Boaosen Bio (Beijing, China). The Mini kit for RNA extraction was purchased from QIAGEN (Düsseldorf, Germany). The SYBR Green Master Mix kits were purchased from TaKaRa (Dalian, China). The DMEM and fetal bovine serum (FBS) were purchased from Thermo Fisher (Waltham, MA, USA).

### 2.4. Immunohistochemistry (IHC)

The paraffin sections were dewaxed in xylene, rehydrated, and placed in graded ethanol solutions. The antigens were retrieved by heating the sections at 98 °C for 30 min in citrate (10 mmol/L, pH: 6.0). Next, a 0.3% hydrogen peroxide solution was dripped onto the sections for 20 min to block the endogenous peroxidase activity; it was blocked with 3% BSA for 15 min (no washing) and incubated with an anti-Siglec-15 antibody (1:150) at 4 °C overnight. PBS was used as the negative control. The next day, HRP-conjugated anti-rabbit IgG was added to the sections. DAB was used as the chromogen, and hematoxylin was used for nuclear staining. The sections were dehydrated, cleared, and mounted. All of the sections were evaluated independently by two pathologists. Ten high-powered fields for each slice were selected randomly. The positive rate scores and staining intensity were graded as follows: (I) 0 for positive cells ≤5%, 1 for 6–25%, 2 for 26–50%, 3 for 51–75%, and 4 for >75%. (II) zero for no staining, 1 for weak positive, 2 for medium positive, and 3 for strong positive. The two parts were multiplied to obtain the final score, including the negative (-) (0–1), weak positive (+) (2–3), positive (++) (4–5), and strong positive (+++) (≥6) scores.

### 2.5. Immunofluorescence Assay (IF)

The cells were seeded into a 12-well plate covered with a cell climbing sheet. The cells were fixed with 4% paraformaldehyde when the cells were fully attached and unfolded on a climbing sheet. Triton X-100 (0.05%) was used to permeabilize the cell membrane for 5 min. Then, the cells were blocked with 5% BSA for 1 h (no washing) and incubated with a primary antibody (anti-Siglec-15, 1:50) at 4 °C overnight. The next day, the slices were incubated with the corresponding secondary antibody and were sealed with a mounting medium containing a DAPI dye.

### 2.6. RNA Interference (RNAi) and Cell Transfection

The small interfering RNA (siRNA) against Siglec-15 was synthesized by Ruibo Technology (Guangzhou, China). The three siRNA sequences were as follows: siSiglec-15-A (5′-GCTCATTTGTGAGAACTAA-3′); siSiglec-15-B (5′-CTACGGAGAACTTGCTCAA-3′); and siSiglec-15-C (5′GGCCCAGGAGTCCAATTAT-3′). The cells were seeded into a 6-well plate and then transfected with 50 nmol/L Siglec-15 siRNA (siSiglc-15) or negative control siRNA (NC) with a Hiperfect^®^ Transfection Reagent (QIAGEN, Germany). After 48 h, the cells were collected to perform the subsequent assays.

### 2.7. RT-PCR

The total RNA was extracted from the cells using a Mini kit (QIAGEN, Hilden, Germany) and it was then reverse transcribed into cDNA using a PrimeScript RT Reagent Kit (Takara, Kusatsu, Japan). The RT-PCR was performed by using a 7500 real-time PCR system (Thermo Fisher, Waltham, MA, USA) with SYBR^®^ Green (Takara, Japan). The 2-ΔΔCt was used to determine the mRNA expression. The primers were as follows: Siglec-15 (5′-CAGCCACCAACATCCATTTC-3′; 5′-CGCTCAAGCTAATGCGTGTA-3′) and β-actin (5′-ctccatcctggcctcgctgt-3′; (5′-gctgtcaccttcaccgttcc-3′).

### 2.8. Western Blotting

The cells were lysed for 40 min using an RIPA buffer (Sigma, China) with a protease inhibitor (Roche, Basel, Switzerland) on ice and then centrifuged at 12,000× *g* at 4 °C for 15 min. The BCA protein assay kit (Thermo Fisher, USA) was used to analyze the total protein concentrations. The total proteins were mixed with 1/4 5× loading buffer at 98 °C for 15 min. The proteins were separated by 12% SDS-PAGE (Sigma, China) and transferred to PVDF membranes (Millipore, Burlington, MA, USA). The anti-Siglec-15 antibody was incubated with PVDF membranes at 4 °C overnight after the blocking with 5% non-fat milk. The next day, the membranes were incubated with an HRP-conjugated secondary antibody. A chemiluminescence detection kit (Sigma, China) was used to detect the protein bands. GAPDH was used as an endogenous control.

### 2.9. Cell Proliferation and Colony Formation Assays

The cells were seeded into a 96-well plate with 2.5 × 10^3^ cells per well (100 mL) and were then observed at 24 h, 48 h, 72 h, and 96 h. At the corresponding times, MTS at a dilution of 1:4 was added to each well. Then the 96-well plate was incubated at 37 °C for 1.5 h. The absorbance value was detected at 492 nm by a microplate reader (PerkinElmer, Waltham, MA, USA). For the colony formation assays, the cells were seeded into a 6-well plate at 1 × 10^3^ cells per well (2 mL) with a high-glucose DMEM for two weeks. The cells were fixed with methyl alcohol for 15 min and stained with 0.1% crystal violet (Sigma, China) for 15 min. Colony numbers containing more than 50 cells were counted.

### 2.10. Wound Healing Assay

The cells were seeded into a 6-well plate at 5 × 10^5^ cells per well and cultured in a high-glucose DMEM containing 15% FBS until they reached an 80–90% confluence. Wounds were created by scratching the cells with a 10 µL pipette tip and then washing the cells twice with PBS. The cells were incubated in a high-glucose DMEM, containing 3% FBS, at 37 °C in a 5% CO_2_ incubator for 3 days. At 24 h, 48 h, and 72 h, images were taken under an inverted light microscope.

### 2.11. Transwell Assays

Twenty-four-well Boyden chambers (Corning, New York, NY, USA) coated with or without Matrigel (Corning, USA) were used to evaluate the cell migration and invasion. A total of 3 × 10^4^ cells in a high-glucose DMEM containing 3% FBS were added to the upper chamber of the wells, and 600 µL of a high-glucose DMEM containing 15% FBS was added to the lower chamber of the wells. After 48 h of incubation at 37 °C, the migratory cells were fixed in methyl alcohol for 15 min and stained with 0.1% crystal violet for 15 min. The cells were counted in more than 3 random fields for each chamber.

### 2.12. RNA Sequencing Assay

The total RNA was extracted by the TRIzol reagent (Thermo Fisher, USA) from cells in the siSiglec-15 group and the NC group. The expression profiles were obtained using an Illumina NovaSeq 6000 according to the manufacturer’s protocols. The FPKM values of the genes were calculated. The threshold values of the differentially expressed genes (DEGs) were defined as fold changes >2 and a *p* < 0.05. The Kyoto Encyclopedia of Genes and Genomes (KEGG) analysis and Gene Ontology (GO) analysis were further performed to interpret the biological significance of the DEGs. The statistical significance of the pathway correlations was determined by the enrichment score.

### 2.13. STRING Analysis and MCODE Analysis

A protein–protein interaction network of DEGs was constructed using the Retrieval of Interacting Genes (STRING, http://string-db.org, accessed on 12 June 2021) (version 11.0, Heidelberg, Germany). The protein nodes that had no interaction with other proteins were removed. The minimum interaction score was defined as 0.09. The key modules and genes were visualized with MCODE in Cytoscape. The top three modules were shown by STRING in Cytoscape. The criteria for the selection of the key genes were as follows: MCODE scores >5.

### 2.14. Statistical Analysis

All assays were repeated three times. A t-test was used to compare the mean between 2 groups, and a one-way analysis of variance was used to compare 3 or more groups. The chi-square test was used to determine the association between Siglec-15 expression and the clinical parameters. Spearman’s analysis was used to determine the correlation between Siglec-15 expression and the pathological parameters. All of the results were analyzed with SPSS software version 21.0 (Chicago, IL, USA) and the graphs were created with GraphPad Prism software 8.0 (San Diego, CA, USA). A value of *p* < 0.05 was considered to be statistically significant.

### 2.15. Ethical Approval

The studies involving human participants were reviewed and approved by the Ethics Committee of the Third Hospital of Hebei Medical University, as described in the Methods section in more detail. The patients provided their written informed consent to participate in this study. All methods were performed in accordance with the relevant guidelines and regulations.

## 3. Results

### 3.1. Expression of Siglec-15 in Human GCTB Tissues and Stromal Cells

**Figure 2:**
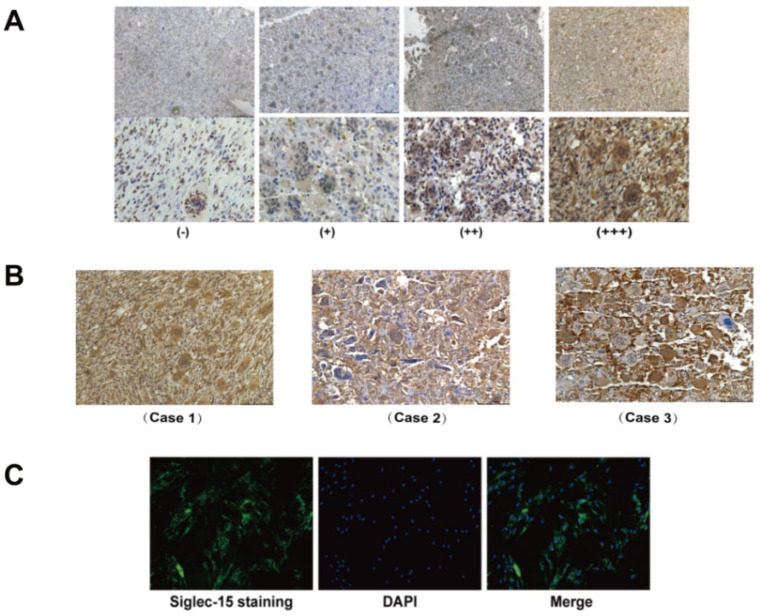
Expression of Siglec-15 in human GCTB tissues and stromal cells. (**A**) Siglec-15 was expressed in the cytoplasm and membrane of GCTB tissues as shown by immunohistochemistry assay. (**B**) Expression of Siglec-5 in recurrent GCTB cases. Case 1: recurrence once, Case 2: recurrence twice, and Case 3: recurrence three times. (**C**) Siglec-15 protein expression in the cytoplasm and membrane of GCTB stromal cells as shown by immunofluorescence assay. Scale bars, 200 µm and 400 µm.

The IHC staining showed that Siglec-15 was mainly localized to the cytoplasm and the membrane of the tumor stroma cells and osteoclast-like multinucleated giant cells (Figure 2A). Moreover, the highest recurrence was three times in our GCTB specimens. The IHC staining showed that there was a positive relation between the recurrence times and the Siglec-15 dyeing strength in the GCTB stroma cells. Here, we showed three recurrent cases: Case 1: recurrence once, Case 2: recurrence twice, and Case 3: recurrence three times (Figure 2B). To further detect the correlation between the Siglec-15 expression and clinical parameters, we evaluated the Siglec-15 staining scores. In the analysis between the Siglec-15 expression and clinical parameters, we found that Siglec-15 showed a significant difference in Campanacci staging and tumor recurrence (*p* < 0.05), whereas it was not significant for sex, age, location, or size (see Table 1). In the analysis between the Siglec-15 expression and pathological parameters, we found that the Siglec-15 expression was significantly correlated with Ki-67 staining, while there was no correlation between the Siglec-15 expression and CD163, CD34, and CD68 (see Table 2). To detect the expression of the Siglec-15 protein in the GCTB tumor cells, we used an IF assay to determine that the Siglec-15 protein was mainly expressed in the cytoplasm and membrane of Hs737. T cells (Figure 2C). Additionally, we detected a high expression of Siglec-15 mRNA in Hs737. T cells by the RT-PCR (Siglec-15/β-actin = 1.636 ± 0.012). Thus, our data suggest that a high Siglec-15 expression in a GCTB is involved in tumor malignancy.

### 3.2. Knockdown of Siglec-15 Expression in GCTB Stromal Cells

**Figure 3:**
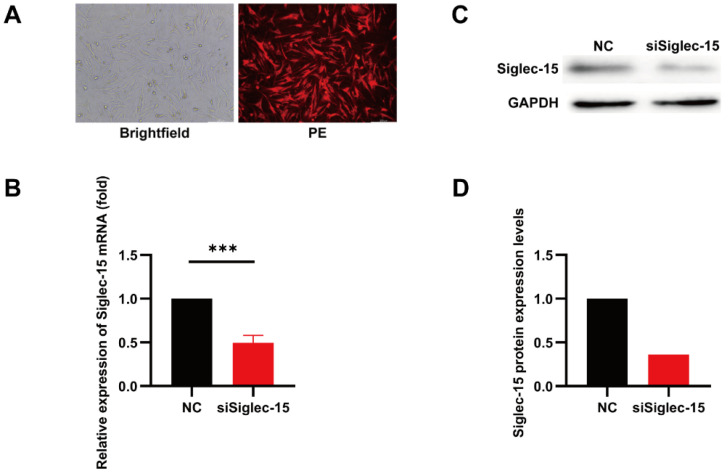
Knockdown of Siglec-15 expression in GCTB stromal cells. (**A**) The transfection efficiency was observed under a fluorescence microscope. (**B**) The silencing efficiency was determined by RT-PCR assay. (**C**,**D**) The silencing efficiency was determined by Western blotting. *** *p* < 0.01. Scale bars, 200 µm.

To examine the influence of Siglec-15 on the biological characteristics of the GCTB tumor cells, we transfected Siglec-15 siRNA and NC siRNA into Hs737. T cells. The transfection efficiency was observed under a fluorescence microscope (Figure 3A). The silencing efficiency of siRNA was determined by an RT-PCR and Western blotting. The results showed that the Siglec-15 mRNA levels and protein levels significantly decreased in the siSiglec-15 group compared with the NC group (Figure 3B–D). Therefore, Siglec-15 siRNA suppresses Siglec-15 expression at the mRNA and protein levels.

### 3.3. Knockdown of Siglec-15 Expression Decreases the Proliferation and Clonal Formation of Hs737. T Cells

**Figure 4:**
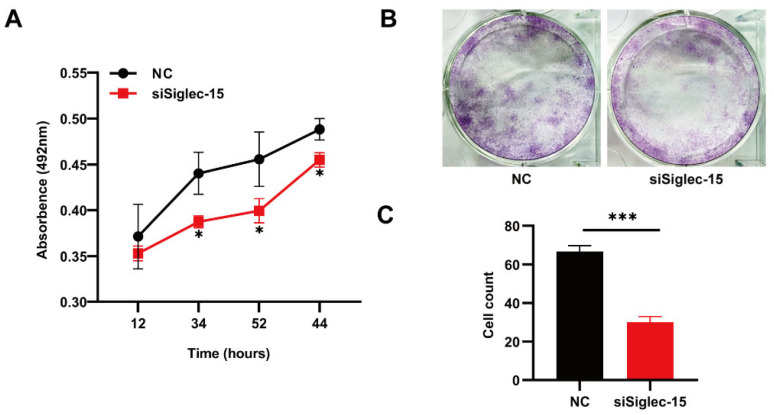
The effect of Siglec-15 silencing on the proliferation and colony formation of GCTB stromal cells. (**A**) The viability of Hs737.T cells transfected with siRNA were detected by the MTS method. (**B**,**C**) Clone experiments showed colony formation of Hs737. T cells. The numbers of Hs737. T cell clones in the siSiglec-15 group were significantly fewer than those in the NC group. * *p* < 0.05, *** *p* < 0.001.

To investigate the role of Siglec-15 in the growth ability of a GCTB, we performed an MTS assay to detect the effects of Siglec-15 expression on the proliferation rate of Hs737. T cells. The results showed that the proliferation rates of the siSiglec-15 group were significantly lower than those of the NC group at 48 h, 72 h, and 96 h, suggesting that the Siglec-15 expression might promote cell proliferation in human GCTB (Figure 4A). In addition, we examined the effect of Siglec-15 expression on the colony formation ability of Hs737. T cells by a colony formation assay. The results showed that the clone numbers of the siSiglec-15 group were significantly less than those of the NC group after 14 days, suggesting that the Siglec-15 expression might promote a colony formation ability in human GCTB (Figure 4B,C).

### 3.4. Knockdown of Siglec-15 Expression Decreases the Migration and Invasion of Hs737. T Cells

**Figure 5:**
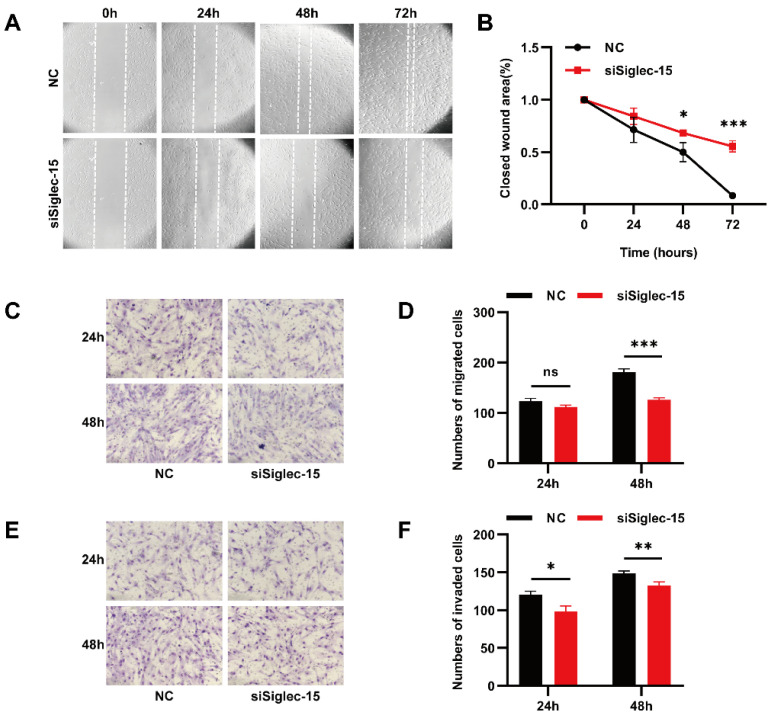
The effect of Siglec-15 silencing on the migration and invasion of GCTB stromal cells. (**A**) Scratch assay showed the mobility of Hs737. T cells transfected with siSiglec-15 compared with the NC group. (**B**) The remaining area was obviously wider in the siSiglec-15 group than in the NC group at 48 h and 72 h. (**C**,**D**) Transwell migration assays showed decreased migration of Hs737 T cells transfected with siSiglec-15 at 48 h compared with the NC group. (**E**,**F**) Transwell invasion assays showed the decreased invasion ability of Hs737. T cells transfected with siSiglec-15 at 24 h and 48 h compared with the NC group. Scale bars, 200 µm, * *p* < 0.05, ** *p* < 0.01, *** *p* < 0.001. ns means no significance.

To investigate the role of Siglec-15 in the migration ability of GCTB stromal cells, we further examined the influence of the Siglec-15 expression on the migration of Hs737. T cells by a wound healing assay. The results showed that the cell-free area of the siSiglec-15 group was clearly wider than that of the NC group at 24 h, 48 h, and 72 h, suggesting that the migration ability of Hs737. T cells decreased in the siSiglec-15 group compared with the NC group (Figure 5A,B). In the transwell migration assay, the number of migrating cells was lower in the siSiglec-15 group than in the NC group at 48 h (Figure 5C,D). These results suggest that the Siglec-15 expression might promote the migration for tumor progression in a human GCTB.

To investigate the role of Siglec-15 in the invasion ability of GCTB stromal cells, we examined the influence of the Siglec-15 expression on the invasion of Hs737. T cells. by a transwell invasion assay. The results showed that the number of cells passing through the Matrigel was significantly lower in the siSiglec-15 group than in the NC group at 24 h and 48 h, suggesting that the Siglec-15 expression might promote an invasion in a human GCTB (Figure 5E,F).

### 3.5. RNA Sequencing Analysis

**Figure 6:**
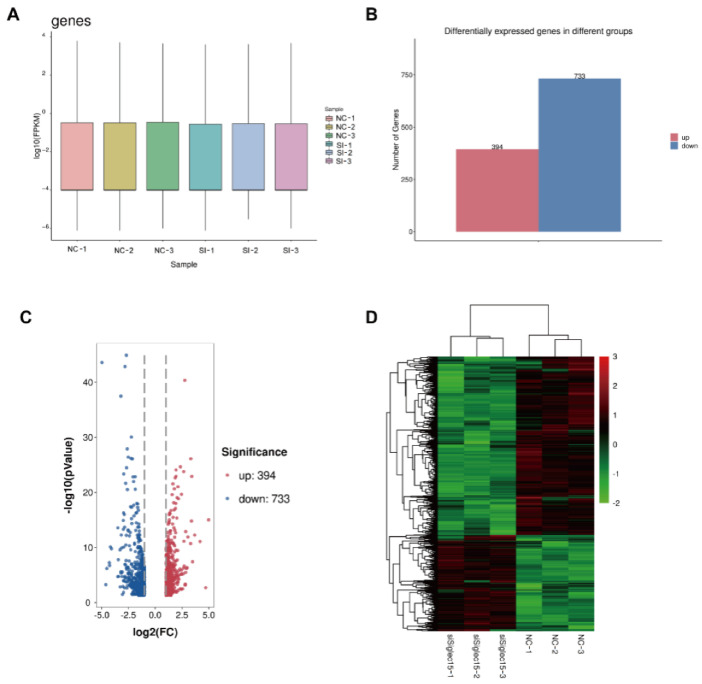
RNA-seq analysis in GCTB stromal cells with Siglec-15 knockdown. (**A**) The RNA intensities for samples were nearly the same after normalization by box plots. (**B**) The differentially expressed genes between the siRNA group and NC group are shown by a histogram. (**C**) The distribution of differentially expressed genes between the NC group and siRNA group is shown by a volcano diagram. (**D**) Highly consistent transcriptional changes were exhibited by heatmaps with three replicates of the NC group and siRNA group.

To explore the mechanism related to the functions of GCTB stromal cells by Siglec-15, we performed a transcriptome analysis by RNA sequencing using three biological replicates in the NC group and the siSiglec-15 group. We identified 1128 DEGs between the NC group and the siRNA group. The box plots showed that the RNA intensities for the samples were nearly the same after normalization (Figure 6A). The differences in gene expression between the NC group and siRNA group are shown in the histogram (Figure 6B). The distribution of the DEGs between the NC group and siRNA group is shown in a volcano diagram (Figure 6C). The highly consistent transcriptional changes were shown by heatmaps with three replicates of the NC group and siRNA group (Figure 6D).

### 3.6. GO Enrichment, KEGG Pathway, STRING, and MCODE Analyses

**Figure 7:**
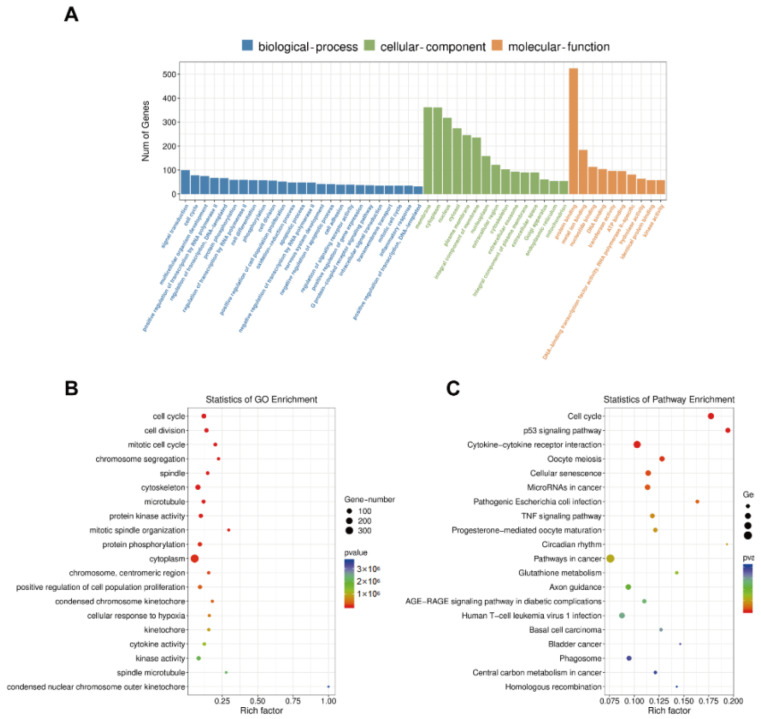
GO enrichment analysis and KEGG pathway analysis. (**A**,**B**) Biological processes, cellular components, and molecular functions were involved in the biological functions of GCTB stromal cells with Siglec-15 knockdown in GO enrichment analysis. (**C**) Twenty signaling pathways linked to cancer were shown by KEGG pathway analysis.

**Figure 8:**
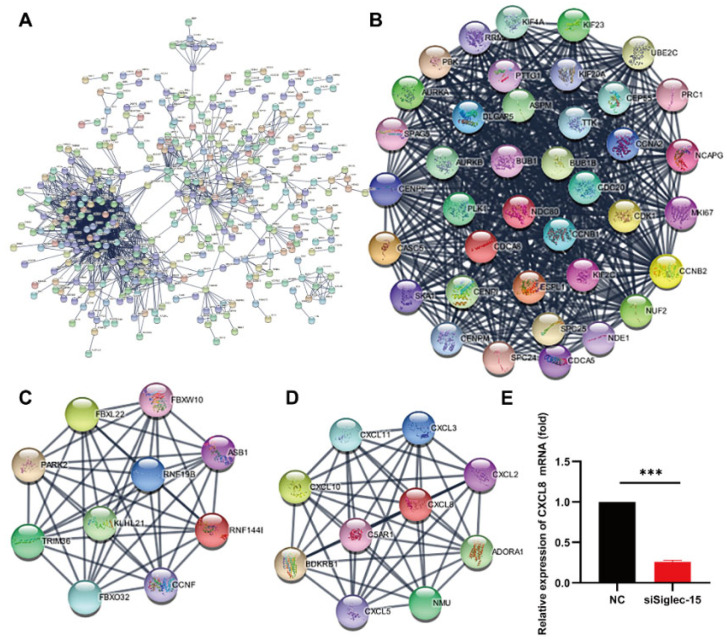
CXCL8 as a potential gene downstream of Siglec-15 in GCTB stromal cells. (**A**) The protein-protein interaction network by STRING. (**B**–**D**) There were 40 genes in module 1, 11 genes in module 2, and 11 genes in module 3 with the density of nodes. (**E**) CXCL8 was downregulated in Hs737. T cells with Siglec-15 knockdown by RT-PCR. *** *p* < 0.001.

The biological processes, cellular components, and molecular functions in the GO enrichment analysis were assessed to identify the biological functions of the genes affected by the Siglec-15 knockdown (Figure 7A). The Siglec-15 knockdown was associated with components of the cell cycle, cell division, mitotic cell cycle, chromosome segregation, spindles, and so forth (Figure 7B). The KEGG pathway analysis showed that the gene changes were linked to cancer, including the cell cycle, p53 signaling pathway, cytokine–cytokine receptor interaction, oocyte meiosis, and so forth (Figure 7C). The top three cancer-associated pathways involving significant genes after the occurrence of the Siglec-15 knockdown included the cell cycle (22 genes), p53 signaling pathway (14 genes), and cytokine–cytokine receptor interaction (30 genes). We speculated that the cell cycle and cytokine–cytokine receptor interaction might play important roles in the progression of the GCTB by the Siglec-15 knockdown. To further identify significantly associated downstream genes, we performed a protein–protein interaction network by a STRING database analysis. A network with 968 nodes and 6545 edges was built with the minimum required interaction score of 0.9 (Figure 8A). In STRING analysis, a total of 62 key genes in the top three modules were identified with an MCODE score ≥ 5. There were 40 genes in module 1, 11 genes in module 2, and 11 genes in module 3 within the density of the nodes (Figure 8B–D).

### 3.7. CXCL8 as a Potential Target Regulated by Siglec-15 in Hs737. T Cells

By a combined validation method using the KEGG analysis, MCODE analysis, and the FPKM value, CXCL8 was selected and was predicted to be an effective potential target of Siglec-15 in Hs737. T cells. CXCL8 had a strong relationship with various cell processes, such as the cytokine-mediated signaling pathway, intracellular signal transduction, IL-17 signaling pathway, and NF-kappa B signaling pathway. The RT-PCR analysis further confirmed that CXCL8 was downregulated significantly upon the Siglec-15 knockdown (Figure 8E). Many studies have indicated that the CXCL family plays critical roles in the regulation of cancer development [29,30,31]. To identify the downstream genes linked to CXCL8, we found the nine highest-linked candidate genes (CXCL2, CXCL3, CXCL5, CXCL10, CXCL11, ADORA1, BDKRB1, C5AR1, and NMU) in module 2 (Figure 8D). All of the genes were confirmed to be closely associated with different kinds of tumor development [31,32,33,34,35,36,37,38]. Overall, the results showed that CXCL8 might play a crucial role in Siglec-15-induced GCTB progression.

## 4. Discussion

Tumor immunotherapy has the potential to reduce tumor malignancy, such as the growth, recurrence, and metastasis of tumors, via a single target or combined targets that inhibit tumor cells or promote immune cells [39,40]. Siglecs has been reported to play critical roles in various diseases, such as infection, osteoporosis, neuropathy, autoimmune disease, and tumor development [41,42,43,44]. In 2001, Siglec-15 was identified as a new member of Siglecs shown to be involved in the development of many tumors [45]. Yoshiharu et al., via the TMHMM program, first reported the expression of Siglec-15 and also reported how it played critical roles in a GCTB [26]. In this study, our results showed that Siglec-15 participated in the GCTB progression and development. The expression of Siglec-15 was closely correlated with Campanacci staging, tumor recurrence, and Ki-67 expression. Furthermore, there is a positive correlation between the GCTB recurrence times and Siglec-15 levels in GCTB stroma cells. The high Siglec-15 expression negatively correlated with the patient’s prognosis, which is consistent with Yoshiharu’s prediction and our previous studies [25,26]. By Real-time PCR and Western blot analysis, we found that the mRNA and protein of Siglec-15 were highly expressed in GCTB stromal cells in vitro. The functional experiments further showed that the proliferation, migration, and invasion of GCTB stromal cells were decreased, suggesting that Siglec-15 plays a critical role in regulating the recurrence and metastasis. Thus, the results indicated that Siglec-15 levels may be as an important prediction for the outcome of GCTB patients.

The aggressive osteolysis also indicates a poor prognosis in GCTB patients [46,47]. In the GCTB progression and development, tumor stromal cells are the main perpetrators and disrupt the balance of the local microenvironment, including in osteoclast-like multinucleated giant cells disorders and immunity inhibition [48,49]. Previous studies have showed that the RANKL-RANK-OPG pathway played an important role in the functional process of osteoclast-like multinucleated giant cells in a GCTB [25]. RANKL overexpression by GCTB stromal cells can induce monocytes expressing RANK to form a large number of osteoclast-like multinucleated giant cells [19]. As an antibody against RANKL, the denosumab was developed and applied clinically and widely, which now has obtained an appreciable treatment effect [14]. Moreover, it is reported that the Siglec-15 was also upregulated during the process of the osteoclast differentiation through the RANKL-RANK-DAP12 pathway [24,25]. The DAP12 is an important receptor for the interaction between Siglec-15 and osteoclast [24]. In our IHC, the high Siglec-15 expression was found in osteoclast-like multinucleated giant cells, further verifying that Siglec-15 directly participated in the process of the formation and differentiation of osteoclast-like multinucleated giant cells. This suggests that Siglec-15 is a duplex and ideal gene for GCTB treatment, including tumor inhibition and osteolytic antagonism. Thus, the inhibition of Siglec-15 expression not only can reduce the malignant progression of GCTB but can be a largely effective target to come in the future for a combined action with denosumab for a further GCTB impediment.

Chemokines are a superfamily composed of 4 subfamilies, C, CC, CXC, and CX3C, which bind to activate the family of G-protein-coupled cell-surface receptors [50]. Previous studies have shown that chemokines participate in numerous biological processes, such as bone fracture, immune response, and cancer [51,52,53]. In cancer, chemokines can mediate cell trafficking in an autocrine or paracrine manner [54]. To further investigate the Siglec-15 downstream pathways in the GCTB, we performed an RNA-sequencing assay. We identified CXCL8 as a downstream gene participating in the Siglec-15-induced GCTB progression. CXCL8 regulates the biological functions in various cancers, such as breast cancer [55], prostate cancer [56], osteosarcoma [57], and colorectal carcinoma [58]. In addition, we further predicted genes linked to CXCL8 genes by STRING analysis and found that CXCL2, CXCL3, CXCL5, CXCL10, CXCL11, ADORA1, BDKRB1, C5AR1, and NMU were closely related to CXCL8 genes.

CXCL2/CXCR1/2, CXCL3/CXCR1/2, CXCL5/CXCR1/2, CXCL10/CXCR3, and CXCL11/CXCR3 are proinflammatory chemokines. In bladder cancer, CXCL2 induces the migration of myeloid-derived suppressor cells (MDSCs) into the tumor microenvironment [59]. In ovarian cancer, Snail promotes the MDSC process via CXCR2 [60]. A high CXCL2 expression is correlated with the tumor stage and patient prognosis [61]. Previous studies have suggested that CXCL3 can enhance the proliferation and migration of prostate cancer [62], breast cancer [63], and colorectal cancer [64]. CXCL5 promotes the migration and invasion of gastric cancer cells by inducing the tumor EMT [65]. CXCL5 in cancer-associated fibroblasts promotes PD-L1 expression to enhance immune inhibition. CXCL10 was significantly upregulated in hepatocellular carcinoma and melanoma brain metastasis [66,67]. CXCL11 expression promoted the migration and invasion of human breast cancer cells and was positively correlated with the overall survival of lung cancer patients [68,69]. Together, the CXCL subfamily plays an important role in cancer development.

ADORA1, an adenosine A1 receptor, is a G-protein-coupled receptor family member that binds adenosine to activate cancer downstream signaling cascades [70]. In hepatocellular carcinoma cells, ADORA1 increased the tumor proliferation and invasion ability, and a high ADORA1 expression predicted a poor survival rate in patients [71]. The combination of PD-1 and ADORA1 antibodies enhanced the treatment efficacy in melanoma and NSCLC mouse models [72]. BDKRB1, a bradykinin B1 receptor, is a receptor bradykinin expressed in the human retina and activated by bradykinin to prompt a calcium influx [73]. In human U87 MG glioblastoma cells, silencing BDKRB1 reduced tumor migration and invasion [74]. C5aR1, a complement component 5a receptor 1, mediates a strong chemoattraction and myeloid cell activation [75]. The C5a-C5aR1 axis recruits MDSCs into the tumor area to further inhibit the cytotoxic T cell function [76]. In C5aR1^-/-^ mice with melanoma, the tumor volume, MDSCs, and regulatory T cells were decreased [77]. Moreover, the combination of the C5a and PD-1 blockade significantly reduced tumor growth and metastasis and prolonged mouse survival [78]. NMU, neuromedin U, is a secreted neuropeptide from the porcine spinal cord [79]. NMU was associated with a malignant grade and poor prognosis in pancreatic cancer and endometrial cancer [80,81]. Overexpressed YAP1 induced NMU expression, which promoted tumor metastasis in vitro and in vivo [80].

## 5. Conclusions

In summary, our study indicated that Siglec-15 plays a critical role in GCTB progression and development. High Siglec-15 levels were significantly associated with a poor prognosis of GCTB patients. Moreover, the proliferation, migration, and invasion of GCTB stromal cells decreased when the Siglec-15 gene was knocked down. In addition, through RNA sequencing analysis, we predicted that CXCL8 may be a gene downstream of Siglec-15 and may be associated with the CXCL2, CXCL3, CXCL5, CXCL10, CXCL11, ADORA1, BDKRB1, C5AR1, and NMU genes. Therefore, the Siglec-15-CXCL8 axis plays an important role in the GCTB development and may provide a new target for GCTB immunotherapy.

## Figures and Tables

**Table 1:** Difference between Siglec-15 expression on GCTB tissues and Clinical parameters.

Clinical Parameters		Cases	Siglec-15 Expression				*p* Value
			(Neg)	(+)	(++)	(+++)	
Gender	Male	20	2	9	8	1	0.091
	Female	36	3	11	11	11	
Age (years)	≤20	4	2	1	0	1	0.126
	20–40	34	3	13	13	5	
	>40	18	0	6	6	6	
Site	Femur	20	0	8	9	3	0.657
	Tibia	12	1	4	3	4	
	Others	24	4	8	7	5	
Tumor size (cm)	<5	30	4	8	9	9	0.383
	≥5	26	1	12	10	3	
Campanacci’s grade	I	15	4	5	4	2	0.018
	II	24	0	12	10	2	
	III	17	1	3	5	8	
Recurrence	Yes	8	0	0	5	3	0.015
	No	48	5	20	14	9	

**Table 2:** Relationship between Siglec-15 expression on GCTB tissues and pathological parameters.

Pathological Parameters	Cases	Siglec-15 Expression				r	*p* Value
		(Neg)	(+)	(++)	(+++)		
CD163 (+)	18	1	10	6	1	−0.334	0.119
CD163 (-)	5	4	0	0	1		
CD34 (+)	12	1	5	4	2	−0.377	0.076
CD34 (-)	15	4	8	2	0		
CD68 (+)	22	5	10	5	2	0.173	0.429
CD68 (-)	1	0	0	1	0		
Ki-67 ≤ 5%	18	2	8	6	2	−0.462	0.027
Ki-67 > 5%	5	3	2	0	0		

## Data Availability

The datasets used and/or analyzed during the current study are available from the corresponding author upon reasonable request.
